# Person-centred care in pharmacy: time to walk the talk

**DOI:** 10.1080/20523211.2025.2511152

**Published:** 2025-05-30

**Authors:** Tarik Al-Diery, Farhat Naz Hussain

**Affiliations:** College of Pharmacy, QU Health, Qatar University, Doha, Qatar

**Keywords:** Person-centred care, patient-centred care, professional identity, metacognition, interprofessional collaboration

## Abstract

Person-centred care is widely recognised as a cornerstone of collaborative practice, which emphasises the patient at the centre of the larger interprofessional team. However, emerging research continues to highlight gaps in the pharmacy professions’ ability to practice person-centred care, owning to ambiguities in its understandings, as well as a risk-averse culture that positions pharmacists as gatekeepers of medication information rather than as proactive collaborators who engage and co-design care with patients. To meet the evolving needs of the healthcare sector, pharmacists must embed person-centred care as a defining element of their professional identity. This requires strengthening foundational education and training, fostering self-reflective and self-awareness practice, and rethinking organisational processes through targeted advocacy that affirms the pharmacist’s integral role within multidisciplinary teams. By doing so, pharmacy can more effectively contribute to optimising health outcomes and delivering truly person-centred care.

## Introduction

The concept of patient-centred care – now more commonly referred to as *person-centered care* – is well established in healthcare discourse (Ekman et al., 2011). Yet its consistent application in pharmacy practice remains inconsistently realised (Burrows et al., 2020). This is due to ambiguities in how it is understood, workplace pressures, and gaps in pharmacist education and training (Burrows et al., 2020; Ng et al., 2021). Defined as healthcare that is respectful of, and responsive to a patient’s preferences, needs, and values, person-centred care is widely acknowledged as a foundation of effective and ethical healthcare (Institute of Medicine, 2001). It evolved in response to dissatisfaction with the biomedical model, which often reduced patients to disease states rather than recognising them as individuals with unique identities, experiences, and goals (Moore et al., 2017). Person-centred care has been at the forefront of collaborative practice where the patient is at the centre of the interprofessional team (Tzelepis et al., 2015).

A systematic review by Scholl and colleagues identified five key dimensions necessary for successful integration of person-centred care: (1) viewing the patient as a unique individual, (2) actively involving patients in their care, (3) providing relevant and personalised information, (4) ensuring effective clinician–patient communication, and (5) fostering patient empowerment.(Scholl et al., 2014) Beyond these dimensions, the personal attributes and characteristics of the healthcare provider – such as empathy, respect for cultural values, and cultural humility – are essential to meaningful engagement (Scholl et al., 2014).

Person-centred care is a multifaceted, relational process requiring skills, knowledge, and attitudes that extend beyond traditional clinical competencies (Park & Choi, 2020). Effective communication, empathy, cultural humility, and the ability to reflect are fundamental to delivering person-centred care (Cavanaugh & Konrad, 2012). For pharmacy to fully embrace this, the profession must move beyond viewing person-centred care as a discrete skill to be deployed in certain scenarios (Wolters et al., 2017). Instead, it must be recognised as a defining identity of the pharmacist and a cornerstone of everyday practice.

Developing person-centred care as a professional identity begins with nurturing metacognitive capabilities – specifically, self-awareness and self-reflection – in both students and practicing pharmacists (Medina et al., 2017). Pharmacists who can recognise their own biases, limitations, and emotional responses are more likely to build rapport with patients and understand their needs more deeply, leading to improved health outcomes for their patients (Medina et al., 2017). While many pharmacy education programmes now acknowledge the importance of non-clinical competencies, the emphasis remains heavily skewed toward developing clinical knowledge and skills (Al-Diery et al., 2024). As a result, ‘soft skills’ essential for meaningful patient relationships are often underdeveloped in pharmacy students and practicing pharmacists (Al-Diery et al., 2024).

Pharmacists are rightly celebrated as medication experts and champions of evidence-based medicine. However, this technical expertise can sometimes eclipse the human dimension of care. When the patient becomes a protocol to follow or a condition to manage, opportunities for authentic connection are lost. Pharmacy education must therefore instil the habit of reflective practice from the undergraduate level, ensuring that person-centred care becomes a lifelong identity, not an occasional initiative (Medina et al., 2017).

Despite growing recognition of its importance, person-centred care is not yet a routine feature of pharmacy practice (Burrows et al., 2020; Ng et al., 2021; Wolters et al., 2017). One key barrier is conceptual ambiguity: the term is widely used but inconsistently understood (Burrows et al., 2020; Wolters et al., 2017). For many pharmacists, person-centred care remains synonymous with communication or information delivery (Burrows et al., 2020). While these are vital components, they do not encompass the full depth of a person-centred approach, which requires shared decision-making, goal setting, and empowering patients to take ownership of their health (Cavanaugh & Konrad, 2012; Scholl et al., 2014).

There is an underlying assumption that educators, students, pharmacists, and professional bodies share a common understanding of what it means to be a person-ceneterd pharmacist (Burrows et al., 2020). However, the literature reveals an absence of a shared understanding amongst pharmacists that undermines implementation efforts (Burrows et al., 2020; Ng et al., 2021; Wolters et al., 2017). Many studies have evaluated the presence or absence of person-centeredness based on isolated interactions (Burrows et al., 2020; Ng et al., 2021; Wolters et al., 2017). These studies frequently show that pharmacists focus on the technical aspects of delivering content by using structured approaches when communicating, often dominating conversations, using closed questions, and delivering generic information (Wolters et al., 2017). This approach neglects the socio-emotional aspects of care, such as empathy, active listening, and eliciting the patient’s unique perspective and concerns.

Research among early-career pharmacists supports these concerns (Burrows et al., 2020). In a longitudinal study by Burrows and colleagues, pharmacy graduates were observed during their internship and first year of practice (Burrows et al., 2020). Despite formal training, many defaulted to transactional, checklist-driven interactions that prioritised efficiency over engagement. Their communication styles reflected the risk-averse and highly structured environments in which they were trained – settings that encourage a gatekeeping role over one of collaboration. Nonetheless, Burrows and colleagues highlighted that person-centred care exists through a continuum of different practices and that pharmacists enact it in a range of ways and to varying extents.

The structural realities of contemporary pharmacy also pose significant challenges. Time constraints, commercial pressures, and outdated expectations continue to frame pharmacists primarily as ‘medication suppliers’. These constraints limit opportunities for deeper, sustained engagement with patients. To truly embrace person-centred care, pharmacists must evolve from passive dispensers to proactive engagers – clinicians who not only educate but also collaborate, advocate, and co-design care with their patients.

To close the gap between aspiration and action, we propose three future-focused strategies for embedding person-centred care in pharmacy:

1. **Reframe person-centred care as a professional identity, not a skill.**
  This requires a shift in mindset. Person-centeredness should be ingrained through reflective learning, meaningful mentorship, and exposure to authentic patient experiences throughout education and into early practice.
2. **Strengthen educational foundations and professional development.**
  Pharmacy schools and Continuing Professional Development programmes must embed person-centred care across the curriculum. This includes teaching metacognitive skills, promoting cultural humility, and normalising reflective practice. Assessment should move beyond technical checklists to evaluating the quality of pharmacist–patient relationships and care planning.
3. **Rethink organisational processes and the pharmacist’s role.**
  Systemic reform is essential. Pharmacists need support and autonomy to engage in person-centred care meaningfully. This includes reengineering workflows, advocating for policies that recognise and reimburse cognitive services, and repositioning pharmacists as integral members of multidisciplinary teams focused on holistic patient outcomes.

We recognise that these changes may not come easily or quickly. Resistance to change, institutional inertia, and competing demands are real challenges. Yet if pharmacists are to be recognised as essential contributors to person-centred healthcare – rather than peripheral dispensers – we must collectively commit to this transformation. A pharmacy profession centred around the person, not just the prescription, is one that empowers patients and enhances our value in an evolving health system.
